# Roles of circRNAs in the tumour microenvironment

**DOI:** 10.1186/s12943-019-1125-9

**Published:** 2020-01-23

**Authors:** Qiuge Zhang, Weiwei Wang, Quanbo Zhou, Chen Chen, Weitang Yuan, Jinbo Liu, Xiaoli Li, Zhenqiang Sun

**Affiliations:** 1 Department of Colorectal Surgery, The First Affiliated Hospital of Zhengzhou University, Zhengzhou, 450052 China; 2 Department of Geriatric Medicine, The First Affiliated Hospital of Zhengzhou University, Zhengzhou, 450052 China; 3 Academy of Medical Sciences, Zhengzhou University, Zhengzhou, 450052 China; 4 Department of Pathology, The First Affiliated Hospital, Zhengzhou University, Zhengzhou, 450052 China

**Keywords:** CircRNAs, Tumour microenvironment, Immunoregulation, Angiogenesis

## Abstract

The tumour microenvironment (TME) constitutes the area surrounding the tumour during its development and has been demonstrated to play roles in cancer-related diseases through crosstalk with tumour cells. Circular RNAs (circRNAs) are a subpopulation of endogenous noncoding RNAs (ncRNAs) that are ubiquitously expressed in eukaryotes and have multiple biological functions in the regulation of cancer onset and progression. An increasing number of studies have shown that circRNAs participate in the multifaceted biological regulation of the TME. However, details on the mechanisms involved have remained elusive until now. In this review, we analyse the effects of circRNAs on the TME from various perspectives, including immune surveillance, angiogenesis, hypoxia, matrix remodelling, exo-circRNAs and chemoradiation resistance. Currently, the enormous potential for circRNA use in targeted therapy and as noninvasive biomarkers have drawn our attention. We emphasize the prospect of targeting circRNAs as an essential strategy to regulate TME, overcome cancer resistance and improve therapeutic outcomes.

## Introduction

During the past decades of cancer research, chemo- and radiotherapy have been recognized as the most effective and extensive approaches for cancer treatment, but their clinical applications are limited due to their toxic side effects [1]. The tumour microenvironment (TME) is the product of the crosstalk between different cell types, and plays a crucial role in the progression, metastasis and therapeutic treatment of cancer [2–4]. Therefore, cancer therapeutic strategies have also gradually shifted from malignant tumour cells to the TME and its complex interactions. In recent years, the TME has been considered a prospective breakthrough in molecular diagnosis and treatment, and TME research has provided new ideas for cancer therapy and possible preventive strategies [5, 6]. Critical issues regarding the roles of the TME in tumour progression and response to treatment include its effects on immunology [7], angiogenesis [8], metastasis and hypoxia [9].

Extensive and in-depth research on the TME should importantly involve noncoding RNAs (ncRNAs), such as microRNAs (miRNAs) [10–12] and long ncRNAs (lncRNAs) [13–15]. With the broad application of high-throughput RNA sequencing (RNA-seq), numerous circular RNAs (circRNAs) have been identified and characterized in humans and other eukaryotes. In recent years, circRNAs have also been involved in TME research. CircRNAs are a class of single-stranded closed circle molecules that lack 5′ and 3′ ends and poly (A) tails, which makes them resistant to RNase R and more stable than linear RNAs [16]. Numerous studies on the distinct properties and diverse cellular functions of circRNAs have revealed their importance in tumourigenesis, reproduction, metastasis, invasion, stem cell regulation and radioresistance, suggesting that circRNAs may potentially serve as required novel biomarkers and therapeutic targets for cancer treatment [17, 18]. Previous studies have focused more on the regulation of tumour parenchyma cells, and recent studies have demonstrated that circRNAs play an essential role in regulating the TME [19, 20].

In this review, we summarize the roles of circRNAs in the TME and lay the foundation for their usability in targeted therapy. In particular, we emphasize the roles of circRNAs in regulating tumour immunity and angiogenesis.

### The tumour microenvironment

Since 1989, and following the description of the “seed and soil theory” hypothesis by Stephen Paget, increasing attention has been paid to the association between cancer and the TME [21, 22], and the TME was deemed the key contributor to tumour proliferation, immune evasion, metastasis and chemoresistance [23]. Accumulating evidence has confirmed that tumour cells must recruit and reprogram the surrounding normal cells to contribute to tumour progression [24]. The TME is a complex scaffold of stromal cells, extracellular matrix (ECM) components, and exosomes [25]. The stromal cells include cancer-associated fibroblasts (CAFs), endothelial cells, pericytes, and immune cells, such as various types of lymphocytes, natural killer (NK) cells, regulatory T cells (Treg), tumour-associated macrophages (TAMs), and myeloid-derived suppressor cells chemokines, matrix metalloproteinases (MMPS), integrins, and other secreted molecules [26]. The dynamic changes in the components described above and other factors related to the TME, such as hypoxia and acidosis, play a significant role in the occurrence, progression and metastasis of tumours (such as triggering an adjustment of the ECM and inducing angiogenesis and immune cell responses in the TME). Different sites and types of tumours have specific TMEs, and the heterogeneity and dynamic changes in the TME lead to cancer therapeutic resistance [27]. Therefore, a thorough understanding of the TME may provide important clues for finding new treatment options and improving the efficacy of treatment.

Over the past few decades, our understanding of TME dynamics has improved exponentially. The TME comprises numerous signaling molecules and pathways that affect angiogenic responses and immune suppression [28]. The approval of antiangiogenic drugs and, more recently, immune checkpoints, by the US Food and Drug Administration (FDA) have reinvigorated the enthusiasm of researchers to understand the role of the TME [29]. Therefore, an in-depth understanding of the molecular mechanisms that regulate tumour angiogenesis and immune suppression may contribute to the development of new therapies that target the dissemination/metastasis of tumour cells.

### CircRNAs

With the rapid development of RNA-seq technologies and bioinformatics, new information regarding circRNAs has gradually been presented. Emerging evidence demonstrates that circRNAs are widespread in eukaryotic cells and have several important properties and numerous biological functions, making circRNAs a focal point of scientific research in the ncRNA field.

CircRNAs have the following special characteristics. (1) Abundance: circRNA expression is widespread in diverse species, such as archaea [30], plants (*Arabidopsis thaliana* and rice) [31, 32], mice [33], zebrafish [34], Drosophila [35] and humans [36]. In addition to their broad expression ranges, approximately one-eighth of the genes expressed in humans produce detectable circRNAs that are expressed at levels more than 10 times higher than the corresponding linear mRNA levels [37, 38]. (2) Stability: due to their covalently closed loop structure and absence of free terminals that confer resistance to RNase R, circRNAs are much more stable than linear RNAs [16, 39]. (3) Conservation: circRNAs exhibit high levels of conservation regardless of the evolutionary distance between species. For example, approximately 15,000 circRNAs are expressed in both human and mouse orthologous loci, representing approximately 15% and 40% of the total circRNAs in humans and mice, respectively [40]. (4) Specificity: in terms of cell type, tissue or developmental stage, circRNAs often show specific expression [41–44].

Numerous circRNA biological functions have been successively clarified with thorough and extensive research, and some are listed here. (1) CircRNAs are specific miRNA “sponges” or “reservoirs”. As competitive endogenous RNAs (ceRNAs), circRNAs contain shared miRNA response elements (MREs) that can interact with target miRNAs. Acting as miRNA sponges, circRNAs can adsorb miRNAs through miRNA binding to the MREs of the circRNAs, thereby preventing miRNAs from complementary pairing with target mRNA 3'-UTR regions; this regulation results in the upregulation of target mRNA expression [45–47]. For example, the most well-known circRNA is CDR1as (antisense to the cerebellar degeneration-related protein 1 transcript, also known as ciRS-7), which contains more than 70 MREs, and it serves as a miR-7 sponge, resulting in reduced miR-7 activity and increased expression of miR-7-targeted transcripts [45]. In addition to acting as specific inhibitors of target miRNAs by functioning as miRNA “sponges”, circRNAs have the opposite ability to stabilize or activate the functions of miRNAs, leading to their designation as so-called miRNA "reservoirs". For example, ciRS-7 is sensitive to miR-671, and the miR-671-dependent Ago2-involved cleavage leads to a disruption of the ciRS-7 and miR-7 association, which ultimately releases miR-7 [48]. Thus, based on the accumulation and storage of miR-7 on ciRS-7, we can consider ciRS-7 to be a miR-7 "reservoir" ready to be activated [48]. Similarly, circHIAT1 protects three of its targets, miR-195-5p/29a-3p/29c-3p, from the inhibition mediated by the androgen receptor (AR); circHIAT1 associates with miRNA binding sites, thereby functioning as a miRNA “reservoir” to stabilize miRNAs and suppress the downstream CDC42 level [49]. (2) CircRNAs interact with RNA-binding proteins (RBPs). RBPs play a central role in gene transcription and translation. The interaction of circRNAs and RBPs is involved in the composition of circRNA functional basis, including circRNA formation, post-transcriptional regulation, and translation [50–53], and such interactions have a role in the execution of circRNA functions [54–56]. (3) CircRNAs act as protein/peptide translators. CircRNAs are reported to function as translation templates, and they may encode proteins/peptides involved in tumour pathogenesis and progress [57, 58]. (4) CircRNAs act as regulators of gene transcription and expression. In addition to regulating gene expression through their role as miRNA sponges, circRNAs also modulate gene expression at transcriptional and posttranscriptional levels [52, 59, 60].

Most aberrantly expressed circRNAs may serve as important regulators of cancer progression through the modulation of numerous cancer hallmarks, functioning to sustain proliferative signalling, promote tumour and antitumour immunity, induce angiogenesis, promote invasion and metastasis, and deregulate cellular energetics [61]. CircRNAs as well as their various functions in the TME are summarized in Table 1.

**Table 1:** Summary of circRNAs and their functions in the TME

Roles	CircRNAs	Origin	Expression	Functions	Targets	References
mediating tumour immune surveillance	circRNAs	exogenous	-	probably activated antitumour immunity	*RIG-I*/ immunocytes	[65]
	circRNAs	exosomes	-	probably regulated antitumour immunity	immunocytes	[58, 70, 72, 73]
	circ-0020397	CRC	up	inhibited the activation and proliferation of T cells, and promoted the viability and invasion of CRC cells	miR-138/ PD-L1	[79]
	circARSP91	HCC	up	enhanced the cytotoxicity of NK cell and upregulated NK-mediated immune responses	*ULBP1*	[92]
	circ-0000977	PC	-	inhibited the killing ability of NK	miR-153 /HIF1α, ADAM10	[168]
promoting angiogenesis	circ0001429	bladder cancer	up	promoted cell metastasis and angiogenesis	miR-205-5p /VEGFA	[99]
	circSCAF11	glioma	up	stimulated angiogenesis and tumourigenesis	miR-421/SP1/ VEGFA	[100]
	circRNA cZNF292	glioma	up	promoted cell proliferation and angiogenesis	VEGFR-1/2, p-VEGFR-1/2 and EGFR	[101]
	circRNA cZNF292	hepatoma	up	promoting cell proliferation, VM, and radioresistance	*SOX9*, Wnt/β-catenin	[103]
	circ-SHKBP1I	GECs	up	stimulated angiogenesis	miR-544a/ FOXP1/ AGGF1 or miR-379/ FOXP2/ AGGF1	[20]
	circ-0010729	HUVECs	up	promoted vascular endothelial cell proliferation	miR-186 /HIF1α	[104]
	circ-002136	GECs	up	stimulated angiogenesis	*FUS*/circ-002136 /miR-138-5p /SOX13 feedback loop	[105]
	circ-DICER1	GECs	up	promoted angiogenesis	MOV10/circ-DICER1 /miR-103a-3p (miR-382-5p) /ZIC4 Hsp90β/PI3K/Akt	[106]
inhibiting angiogenesis	circHIPK3	bladder cancer	down	inhibited cell migration, invasion, and angiogenesis	miR-558/ HPSE/ MMP-9 and VEGF	[107]
	circSMARCA5	GBM	down	inhibited angiogenesis	VEGFA, SRSF1	
	circ-0003575	HUVECs	up	inhibiting angiogenesis	potential circRNA-miRNA-mRNA network	[110]
improving endothelial cell permeability	circRNA IARS	exosomes derived from PC cells	up	enhanced cell invasion, metastasis and endothelial cell permeability	miR-122/ RhoA/ F-actin	[112]
hypoxia	circDENND4C	breast cancer	up	promoted cell proliferation	-	
	circDENND4C	breast cancer	up	promoted cell glycolysis, migration, invasion and proliferation	miR-200b and miR-200c	[116]
	circDENND2A	glioma	up	promoted cell migration and invasion	miR-625-5p	[169]
	circ-0000977	PC	-	mediated immune escape	miR-153/ HIF1α	[168]
	circ-0010729	HUVECs	up	enhanced cell proliferation, migration and suppressed apoptosis	miR-186/ HIF1α	[104]
	circRNA cZNF292	hepatoma	up	promoted cell proliferation, VM, and radioresistance	SOX9, Wnt/β-catenin	[103]
causing ECM remodelling	circ-0000096	gastric cancer	down	affected cell growth and migration	VEGF, MMP-2 and MMP-9	[120]
	circLMNB1	CRC	up	promoted cell dissemination, invasion and EMT	MMP-2 and MMP-9	[121]
	circDENND4C	breast cancer	up	suppressed cell migration and invasion	MMP-2 and MMP-9	[116]
	circ-0007334	PDAC	up	promoted cell invasion	miR-144-3p and miR-577/ COL1A1, MMP-7	[122]
	circRNA cSMARCA5	HCC	down	inhibited cell proliferation and migration	miRNA-17-3p and miRNA-181b-5p/ TIMP-3	[124]
exosomes	circRNA IARS	exosomes derived from PC cells	up	promotedcell invasion and metastasis	miR-122	[112]
	circ-DB	exosomes derived from adipocytes	up	promoted tumourigenesis and metastasis of HCC	miR-34a/ USP7/ cyclin A2	[135]
	ciRS-133 (circ-0010522)	exosomes derived from GC cells	up	promoted white adipose browning in patients with gastric cancer	miR-133/ PRDM16	[119]

### CircRNAs mediate tumour immune surveillance

**Fig. 1:**
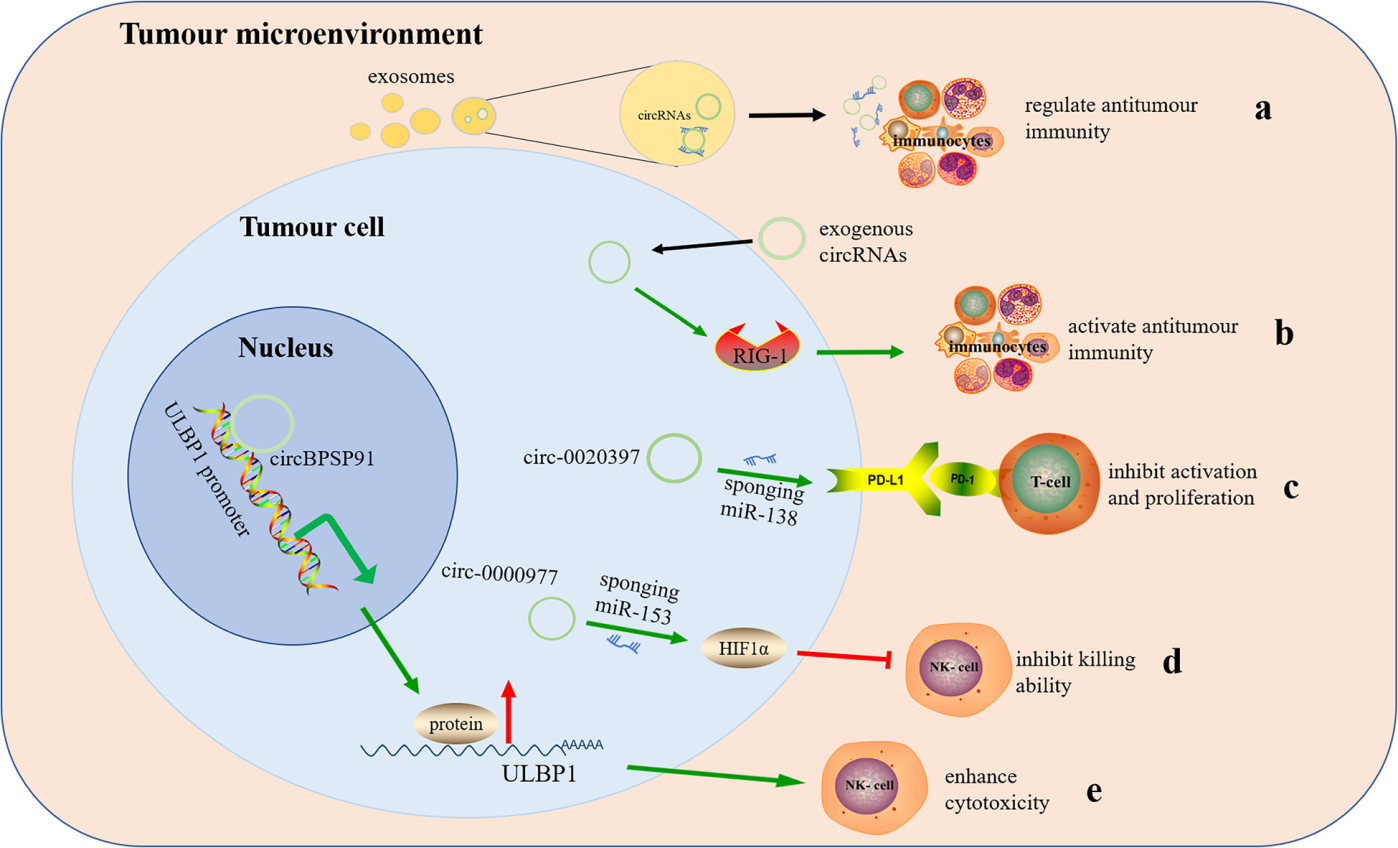
CircRNAs play a vital role in regulating tumour immunity. **a** circRNAs are transported to immunocytes through exosomes and extracellular vesicles secreted by the tumour cell to regulate immune responses. **b** exogenous circRNAs entering tumor cells may activate *RIG-1*-mediated pathway and activate antitumour immunity. **c** circRNAs promote the expression of PD-L1 in tumour cells, inhibit the activation and proliferation of T cells, and induce T cell apoptosis. **d** circRNAs can inhibit the killing ability of NK cells through the circ-0000977/miR-153/HIF1α axis. **e** circRNAs can also upregulate target mRNAs and ULBP1 protein levels to enhance the cytotoxicity of NK cell and upregulate NK-mediated immune responses. The green arrow indicates stimulatory modification, and the red “T” symbol indicates inhibitory modification

It is one of the focuses of immunology research to fully understand the molecular mechanism of the cancer-related immunity that affects cancer development and progression, which may be beneficial for the development of more effective immunotherapeutic strategies. Previous studies have shown that cancer-related immunity plays a dual role in cancer, functioning to both protect the host and promote tumour growth [62, 63]. For example, cancer-related immune responses can protect the host by destroying cancer cells or inhibiting their outgrowth; on the other hand, they can also promote tumour progression by selecting for tumour escape variants or establishing conditions within the TME that facilitate the development of a tumour-specific adaptive immune response [64]. In recent years, circRNAs have been found to play a potential part in regulating tumour immunity. As immune system antigens, exogenously purified circRNAs may mediate the activation of innate immunity by activating the retinoic acid-inducible gene I (*RIG-I*)-mediated pathway *in vitro* [65]. CircRNA-induced nucleic acid sensor *RIG-I* is a well-known innate immunity regulator [66, 67], and *RIG-I* agonists have been shown to activate anticancer immune responses to fight tumours [68, 69]. Therefore, exogenous circRNAs entering tumour cells have the potential to affect *RIG-I* and activate antitumour immunity (Fig. 1b). Studies have shown that tumour cell-derived exosomes are involved in multiple immune activities in tumour progression [58, 70, 71]. In addition, some exosomal RNAs from donor cells, including circRNA, can function in recipient cells [72–74]. These studies indicated that circRNAs may be transported to immunocytes through exosomes and extracellular vesicles (EVs) to regulate immune responses in tumours by functioning as potential tumour antigens (Fig. 1a). For instance, researchers discovered that circRNAs were downregulated and transferred to exosomes from KRAS mutant colon cancer cells [75]. In addition, circRNAs can coprecipitate with EVs, and since EVs can be taken up by other cells, excreted circRNAs may contribute to cell-to-cell communication [76]. Moreover, the plasma expression levels of circRNAs are closely related to the levels of tumour-infiltrating lymphocytes (TILs) in the TME [77]. Therefore, circRNAs have rich potential to regulate tumour immunity.

### CircRNAs regulate immune escape via PD-L1

Tumour immune escape refers to the phenomenon of tumour cells growing and metastasizing via various mechanisms to avoid recognition and attack by the immune system. The mechanism of tumour immune escape includes immunosuppression. Programmed death 1/programmed death-ligand 1 (PD-1/PD-L1), known as an immune checkpoint, is an important component of tumour immunosuppression [78]. One recent study showed that a circRNA acted as a ceRNA to regulate the expression of PD-L1, thereby helping the tumour escape immune surveillance [79]. The interaction between PD-1 and PD-L1 can effectively inhibit the activation of effector T lymphocytes, ultimately leading to tumour immune escape [80]. The inhibitory checkpoint PD-L1 is highly expressed in multiple malignancies [81, 82], so developing drugs that block the PD-L1 pathway is an attractive potential cancer immunotherapy. Currently, drugs targeting PD-L1 are being tested in clinical trials against multiple cancer types, including colorectal cancer (CRC) [83], non-small cell lung cancer (NSCLC) [84, 85], and urothelial carcinoma [86]. In the past few decades, the roles of miRNAs in regulating the expression of the PD-1/PD-L1 immune checkpoint and the sensitivity of tumours to chemotherapy drugs have been well studied [87, 88], revealing that circRNAs contribute to immune escape through a circRNA-miRNA-PD-1/PD-L1 axis (Fig. 1c). For instance, the circRNA circ-0020397 was found to bind to miR-138, suppress miR-138 activity, and consequently promote the expression of miR-138 targets, such as telomerase reverse transcriptase and PD-L1, in CRC cells (CRCCs). Due to the high circ-0020397 expression in CRCCs, PD-L1 is upregulated and can interact with PD-1 to induce T cell apoptosis and inhibit T cell activation and proliferation, leading to cancer immune escape. Studies have shown better clinical efficacy of PD-1/PD-L1 blockers in patients with high PD-L1 expression [79]. Therefore, regulating PD-1/PD-L1 expression by targeting related circRNAs may be a direction of future immune checkpoint therapeutic research.

### CircRNAs regulate the cytotoxicity of natural killer cells

Natural killer (NK) cells are considered the first line of defence for host immune surveillance and play a vital role in antitumour immunotherapy. Because NK cells have no major histocompatibility complex (MHC) limitations on the recognition and destruction of target cells, an increasing number of immunostimulants can be produced to enhance cell killing [89]. Increasing experimental evidence has shown that the activity and density of NK cells in the TME correlates with prognoses in a variety of cancers [90]. Xu reported that the densities of infiltrating NK cells in tumour nests and stroma were significantly associated with patients' postoperative prognoses [91]. Recent studies elucidated another mechanism by which tumour cell-derived circRNAs participate in tumour immune surveillance by enhancing NK cell activity and upregulating NK-mediated immune responses. For instance, in hepatocellular carcinoma (HCC) cells, circARSP91 was reported to enhance the cytotoxicity of NK cells by upregulating the expression of UL16 binding protein 1 (ULBP1) at the mRNA and protein levels (Fig. 1e). In addition to acting as miRNA sponges, circRNAs also regulate the translation of target mRNAs at the posttranscriptional levels. For example, circARSP91 may interact with the *ULBP1* promoter region and recruit RNA polymerase II to enhance the expression of the *ULBP1* gene (Fig. 1e). In this study, ULBP1 was upregulated by circARSP91 and assisted NK cells in identifying and attacking target tumour cells [92]. In addition, a recent study showed that hypoxia induced the expression of circ-0000977 in pancreatic cancer (PC). Circ-0000977 knockdown enhanced the killing effect of NK cells on PC cells under hypoxic conditions through hypoxia-inducible factor 1-alpha (HIF1α). The circ-0000977/miR-153/HIF1α axis modulates the HIF1α-mediated immune escape of PC cells by downregulating the sensitivity to NK cell-mediated lysis [93] (Fig. 1d). Thus, the above studies indicate that circRNAs can modulate the activity or cytotoxicity of immune cells in the TME, thereby mediating tumour immune surveillance.

### CircRNAs regulate angiogenesis and endothelial monolayer permeability

Angiogenesis is a complex process by which new blood vessels are formed from pre-existing vessels by sprouting, remodelling and expanding primary vascular networks [94]. Inducing angiogenesis by influencing the microenvironment is one hallmark of cancer. The growth and metastasis of solid tumours depend on angiogenesis to supply sufficient nutrients and oxygen to cancer cells. The tumour-associated neovasculature plays key roles in multiple aspects of tumour biology, including tumour dissemination/metastasis, metabolic deregulation and cancer stem cell (CSC) maintenance, and current research suggests that circRNAs mediate angiogenesis [20]. In the TME, circRNAs play an essential role in proangiogenic and antiangiogenic signalling networks related to the “angiogenic switch”.

### Heterogeneity of angiogenesis regulated by circRNAs

The majority of human tumours present startling heterogeneity in many of their morphological and physiological features, such as their expression of cell surface receptors and proliferative and angiogenic potential [95]. In recent years, circRNAs have been shown to act as auxiliary diagnostic biomarkers of diverse cancers, and their expression is reported to be heterogeneous in different cancers [96]. Similarly, the latest research suggests that circRNAs are heterogeneous in their regulation of tumour angiogenesis.

### Promoting angiogenesis

**Fig. 2:**
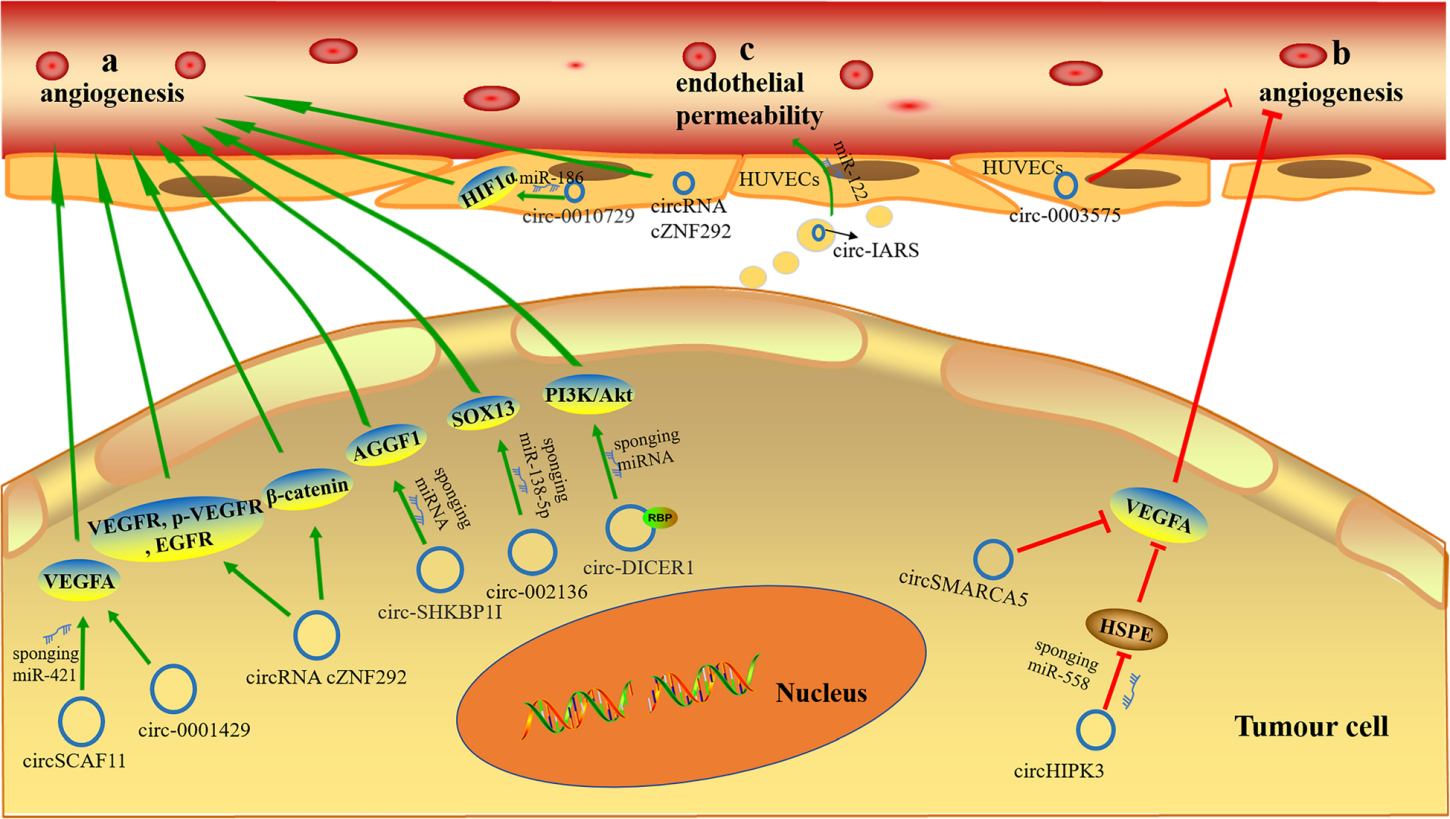
The functional roles of circRNAs in tumour angiogenesis and endothelial monolayer permeability. **a** circRNAs promote angiogenesis. **b** circRNAs inhibit angiogenesis. **c** circRNAs promote endothelial monolayer permeability. The green arrow indicates stimulatory modification, and the red “T” symbol indicates inhibitory modification

On the one hand, circRNAs directly regulate the expression of vascular endothelial growth factor A (VEGFA) (Fig. 2a). For instance, circRNA-MYLK and VEGFA were significantly upregulated and co-expressed in breast cancer. Importantly, overexpressing circRNA-MYLK promoted the tubular structure formation of human umbilical vein endothelial cells (HUVECs) *in vitro* and angiogenesis *in vivo* by upregulating VEGFA [97]. As a member of the growth factor family, VEGFA has a large capacity to stimulate the angiogenic milieu, as it can increases microvascular density and vascular permeability, which promotes tumour angiogenesis and metastasis and leads to the resistance of tumours to antiangiogenic therapy [98]. Similarly, circ0001429 was found to upregulate VEGFA expression by sponging miR-205-5p to promote the growth and metastasis of bladder cancer cells [99]. CircSCAF11 activates the VEGFA transcription via the miR-421/SP1/VEGFA axis, which stimulates angiogenesis and tumourigenesis of glioma [100]. Furthermore, the circRNA cZNF292, generally expressed in a hypoxic environment, was also discovered to be expressed in glioma U87MG and U251 cells [101]. This study showed that silencing circRNA cZNF292 could significantly inhibit the proliferation and angiogenic potential of glioma cells by downregulating the expression of VEGFR-1/2, p-VEGFR-1/2 and EGFR. CircRNAs were demonstrated to be abundantly expressed in endothelial cells, and the circRNAs cZNF292, cAFF1, and cDENND4C were upregulated by hypoxia. Among them, cZNF292, a significantly hypoxia-regulated circRNA, exhibits a proangiogenic function in endothelial cells [102]. Interestingly, while further exploring the mechanism of the angiogenic function of cZNF292, researchers found that cZNF292 had neither a cis-regulatory function in host gene expression nor a putative function as a miRNA sponge [102]. Further investigation showed that cZNF292 knockdown inhibited hepatoma vasculogenic mimicry (VM) and radioresistance *in vitro* and *in vivo* by increasing sex-determining region Y (SRY)-box 9 (*SOX9*) nuclear translocation, subsequently reducing Wnt/β-catenin signalling pathway activity [103].

On the other hand, circRNAs regulate the expression of downstream molecules related to angiogenesis by acting as specific miRNA “sponges” (Fig. 2a). For instance, circ-SHKBP1I was shown to increase the expression of AGGF1 via the miR-544a/FOXP1 or miR-379/FOXP2 pathway, which stimulated glioma angiogenesis [20]. The researchers also utilized loss-of-function experiments in HUVECs to demonstrate that circ-0010729 knockdown suppressed proliferation and migration and enhanced apoptosis. The authors also identified that the crucial regulatory effect of circ-0010729 on vascular endothelial cells was mediated by targeting the miR-186/HIF1α axis [104]. Emerging research indicates that the *FUS*/circ-002136/miR-138-5p/SOX13 feedback loop plays a critical role in promoting glioma angiogenesis. This study found that circ-002136 was highly expressed in glioma-associated endothelial cells (GECs), and silencing circ-002136 inhibited glioma angiogenesis [105].

Additionally, circRNAs also simultaneously act as RBPs and miRNA "sponges" (Fig. 2a). For instance, by binding the RBP MOV10 and acting as a molecular sponge to adsorb miR-103a-3p/miR-382-5p, circ-DICER1 upregulated the expression of ZIC4 and its downstream target Hsp90β. Upregulated circ-DICER1 in GECs promoted angiogenesis by activating the MOV10/circ-DICER1/miR-103a-3p (miR-382-5p)/ZIC4 Hsp90β/PI3K/Akt signalling pathway [106]. Therefore, circRNAs promote angiogenesis through different mechanisms to create a favourable microenvironment for tumour growth and metastasis.

### Inhibiting angiogenesis

In contrast to the above proangiogenic functions, circRNAs also play an essential antiangiogenic role (Fig. 2b). CircHIPK3 was reportedly downregulated in human bladder cancer. Enforced overexpression of circHIPK3 significantly inhibited the migration, invasion, and angiogenesis of bladder cancer cells via sponging miR-558 to suppress the expression of heparanase (HPSE) and its downstream targets MMP-9 and VEGF [107]. Moreover, Davide and colleagues found that circSMARCA5 is an upstream regulator of the pro- to antiangiogenic VEGFA isoform ratio within glioblastoma multiform (GBM) cells and acts as a prospective antiangiogenic molecule. CircSMARCA5 acts as sponge for serine- and arginine-rich splicing factor (SRSF1) to regulate angiogenesis via a physical interaction. Additionally, circSMARCA5 inhibits angiogenesis by inducing VEGFA alternative splicing and decreasing microvascular vessel density [108]. Bevacizumab, a humanized monoclonal antibody against VEGFA, has not produced the expected results in antiangiogenic therapies [109]. CircRNAs as therapeutic targets might be an effective alternative to therapy based on monoclonal anti-VEGFA antibodies. Through loss-of-function experiments, researchers also revealed that silencing circ-0003575 promoted the proliferation and angiogenesis of HUVECs [110].

The above results led to the determination that different circRNAs play multifaceted and even opposite roles in regulating angiogenesis through multiple mechanisms, thus proving the heterogeneity of circRNAs. Therefore, we speculate that in future studies on angiogenesis inhibitors, how circRNAs play an effective targeted therapeutic role to inhibit angiogenesis will be the research focus.

### Endothelial monolayer permeability regulated by circRNAs

In addition to the regulation of angiogenesis, exosome-derived circRNAs can also promote the metastasis and dissemination of cancer cells by regulating the permeability of endothelial cells (Fig. 2c). Tumour metastasis is the main cause of cancer-related death. Tumour endothelial cells, which acquire their specific characteristics in the TME, stimulate the metastasis of tumour cells. In particular, endothelial cells play a crucial role in the initial stage of tumour metastasis [111]. Here, a molecular mechanism by which exosome-derived circRNAs regulate the permeability of endothelial cells in the TME is described. For instance, the circRNA IARS was found to enter HUVECs via exosomes derived from PC cells and enhance endothelial cell permeability by disrupting the tight junction between the endothelium. The above function was achieved through the circRNA IARS/miR-122/RhoA/F-actin molecular pathway. Due to the high level of circ-IARS expression in PC tissues and plasma exosomes of patients with metastatic disease, endothelial monolayer permeability was enhanced, which promoted the formation of a microenvironment suitable for tumour invasion and metastasis [112]. Therefore, circRNAs can be potential targets for endothelial cells in the initial stage of tumour metastasis, helping to prevent early tumour cell metastasis by inhibiting endothelial cell permeability.

### Hypoxia regulates circRNA production

**Fig. 3:**
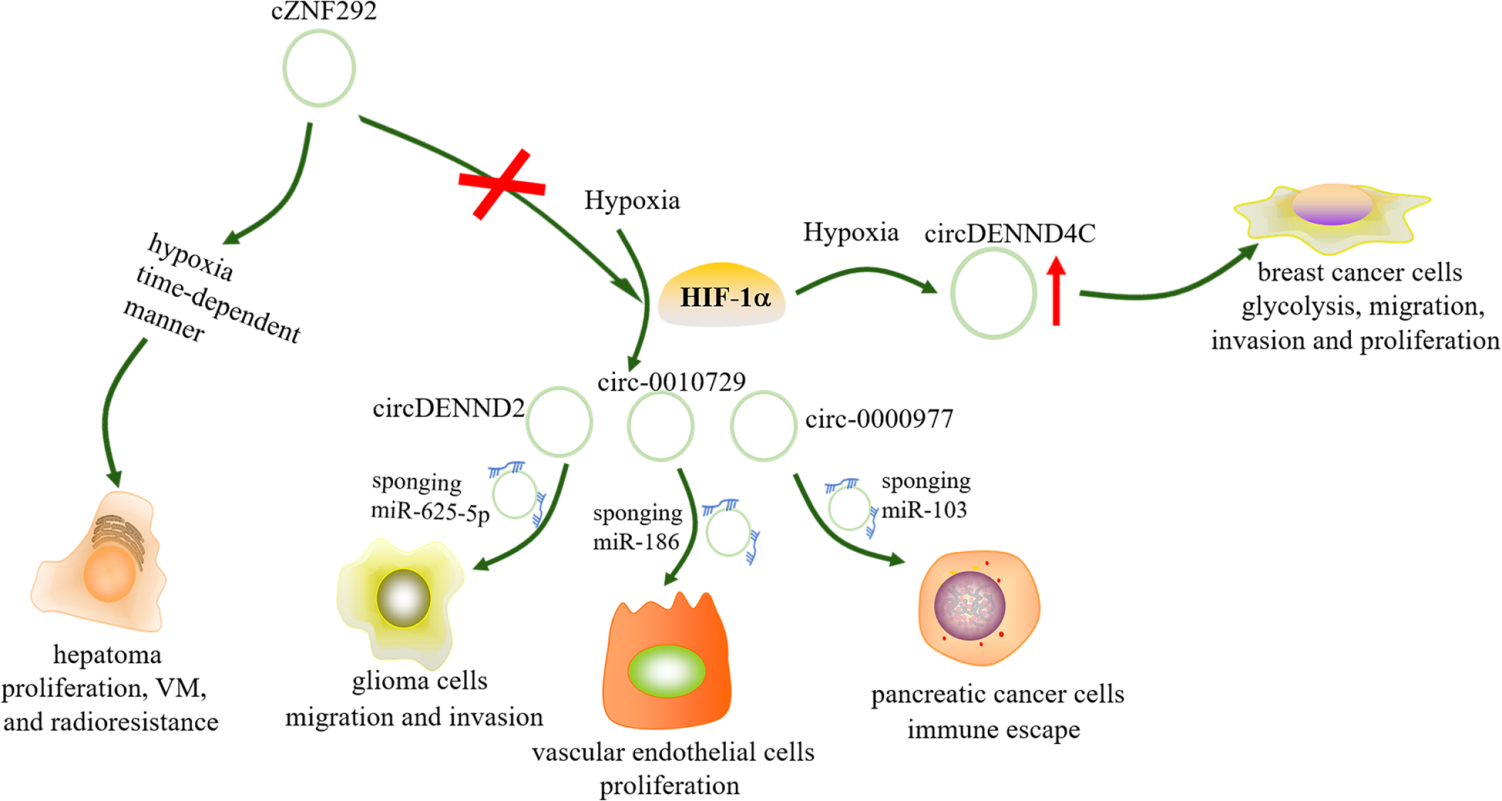
Hypoxia-related circRNAs promote the proliferation, migration and invasion of cancer cells and are also associated with angiogenesis and anti-radiation therapy of cancer. The green arrow indicates stimulatory modification

Hypoxia is a key feature of the TME and has a profound impact on cancer aggressiveness and therapy. The molecular mechanisms of responses to hypoxia are extremely complex. HIFs, transcriptional regulators, play a key role in regulating the responses of the TME and the proliferation and metastasis of cancer cells by activating the transcription of downstream oncogenes containing hypoxia-responsive elements (HREs) and regulating various signal pathways [113, 114]. For instance, circRNAs regulated by hypoxia in endothelial cells were identified for the first time [102]. Subsequently, in breast cancer cells, HIF1α was reported to be crucial for upregulating circDENND4C under hypoxic conditions, indicating that circDENND4C is an HIF1α-associated circRNA and promotes the proliferation of breast cancer cells [115] . Under hypoxia, loss-of-function experiments indicate that circDENND4C knockdown could suppress glycolysis, migration and invasion by increasing miR-200b and miR-200c in breast cancer cells [116]. Another study also showed that hypoxia induced the expression of circDENND2A, which promoted the migration and invasion of glioma cells by sponging miR-625-5p. Via clinical analysis, this study also demonstrated the existence of a circDENND2A/miR-625-5p axis in glioma tissues, which was associated with HIF1α [19]. The hypoxia-induced expression of circ-0000977 and the circ-0000977/miR-153 axis modulates the HIF1α-mediated immune escape of PC cells via the miR-153 downstream target HIF1α [93]. Hypoxia also induced upregulation of circ-0010729, which was shown to regulate vascular endothelial cell proliferation and apoptosis via targeting the miR-186/HIF1α axis [104]. Interestingly, another study indicated that cZNF292 was induced by hypoxia in a time-dependent manner in hepatoma cells independent of HIF1α, promoting hypoxic hepatoma proliferation, VM, and radioresistance [103]. As hypoxic microenvironment-related circRNAs participate in angiogenesis, metastasis, invasion, and resistance to radiation therapy (Fig. 3), circRNAs have great potential to be used as targets and may play crucial roles in blocking many undesirable traits for cancer under hypoxia.

### Remodelling of the extracellular matrix (ECM)

The TME is mainly composed of stromal cells and ECM components. The ECM is a highly dynamic structural network composed of many matrix components that continuously undergo remodelling mediated by several matrix-degrading enzymes in the process of tumourigenesis and development [117].

The ECM is degraded by various proteases, with the MMP family having a pivotal role. These enzymes (MMPs) support tumour cell invasion of the basement membrane and stroma, blood vessel penetration, and metastasis by interacting with macromolecules on the basement membrane to degrade and stimulate ECM remodelling [118]. CircRNAs are reported to be involved in ECM remodelling by regulating the expression of MMPs [116, 119]. For example, in gastric cancer cells, the protein expression levels of VEGF and the migration-related proteins MMP-2 and MMP-9 were significantly decreased after knockdown of circ-0000096, indicating that circ-0000096 may affect cell growth and migration by regulating matrix remodelling and angiogenesis [120]. CircLMNB1, which is highly expressed in CRC, downregulates MMP-2 and MMP-9 expression and inhibits epithelial-mesenchymal transition (EMT) after gene knockdown, thereby affecting tumour dissemination and invasion [121]. Under hypoxic conditions, silencing circDENND4C significantly reduced the migration and invasion of breast cancer cells by downregulating the protein expression levels of MMP-2 and MMP-9 [116]. Remodelling of the ECM and angiogenesis in cancer stroma can be considered part of the tumour invasion processes. Circ-0007334 regulates MMP-7 and collagen type I alpha 1 chain (COL1A1) by competitively adsorbing miR-144-3p and miR-577 to enhance the expression and functions of MMP-7 and COL1A1 in pancreatic ductal adenocarcinoma (PDAC) [122]. COL1A1 is responsible for encoding ECM remodelling-related collagens, which are the most abundant proteins in the ECM. COL1A1 is an individual ECM gene and has been reportedly associated with tumour invasion and metastasis [20].

The activity of these MMPs is tightly regulated in numerous ways, such as by transcriptional regulation, proteolytic activation and interaction with tissue inhibitors of metalloproteinases (TIMPs) [123]. TIMPs are endogenous inhibitors that can inhibit the activity of MMPs to prevent degradation of the ECM, and the local balance between TIMPs and MMPs plays a crucial role in ECM homeostasis. Low expression of circRNA cSMARCA5 in HCC can promote the expression of TIMP-3 by sponging miRNA-17-3p and miRNA-181b-5p and inhibit the proliferation and migration of HCC cells [124]. Therefore, circRNAs play an important role in the metastasis and invasion of cancer by regulating the TME, especially functioning in matrix remodelling. CircRNAs may serve as key targets for tumour detection and treatment and open new directions for future research.

### Exosome-derived circRNAs and the TME

Exosomes are extracellular vesicles 40-200 nm in diameter with a lipid bilayer membrane structure; they are secreted by almost all cell types under both physiological and pathological conditions and are widely found in the microenvironment [125, 126]. Exosomes contain diverse proteins, lipids, DNAs, and RNAs (mRNA, miRNAs, lncRNAs, and circRNAs) [127, 128], which are involved in intercellular communication when they are released and transferred into recipient cells [73, 129, 130]. Intercellular information transmission in the TME is crucial for tumour progression. Numerous studies have shown that abundant and stable circRNAs are present within exosomes [75, 76, 131]. Recently, researchers have revealed that tumour exo-circRNAs may be transported to immunocytes as tumour antigens to activate antitumour immunity or bind to miRNAs and proteins to regulate immunocyte activity. In addition, when exo-circRNAs are transported from tumour cells to immunocytes, they help release the miRNAs into the immunocytes to silence related target genes [132]. Similarly, Bai described two possible main regulatory mechanisms of exo-circRNAs by sponging miRNAs [133]. For example, exosomes derived from PC cells carry circRNA IARS to the target site (HUVECs). Then, the circRNA is absorbed and bound to miR-122, thereby relieving the inhibition of target gene expression [112]. The TME includes adipocytes [3, 134], and one study showed that the exo-circ-deubiquitination (exo-circ-DB) derived from adipocytes upregulated the expression levels of USP7 and Cyclin A2 by sponging miR-34a and activating the USP7/CyclinA2 signalling pathway, thereby promoting the tumorigenesis and metastasis of HCC [135]. In addition, plasma exosome-derived ciRS-133 (circ-0010522) aggravates tumour cachexia and increases oxygen consumption by promoting white adipose browning in patients with gastric cancer [119]. Numerous experiments have demonstrated that tumour-related exosomes (TEXs) play an essential role in TME maturation and cancer progression [136–138]. Because circRNAs are located in exosomes, exo-circRNAs are characterized by a transferable targeting ability as well as by the original biological functions of circRNAs. Therefore, the function and potential application of exo-circRNAs in the TME are worthy of affirmation and may be gradually uncovered by future research.

### The clinical potential of circRNAs

#### CircRNAs regulate cancer chemoradiation resistance

Although chemotherapy and radiotherapy are still the preferred methods for cancer therapy behind surgery, local recurrence and distant metastasis still occur in a considerable fraction of cancer patients due to the development of resistance. Intrinsic and extrinsic factors can affect cancer cell resistance to chemoradiation. The extrinsic factors in the TME that promote chemoradiation resistance and tumour recurrence include hypoxia, the ECM, and the expression of angiogenic markers such as VEGF and HIF-1α [139–142], which may involve circRNAs. Therefore, understanding the regulatory mechanisms of circRNAs involved in radiotherapy and chemotherapy resistance can identify novel targets to optimize therapy.

Recently, studies found that the expression profile of circRNAs was altered in AZD9291-resistant NSCLC cell lines, 5-FU-based chemoradiation-resistant CRC cells, gemcitabine-resistant PC cells, tamoxifen-resistant breast cancer cells and radioresistant oesophageal cancer cells [143–147]. Further analysis revealed that some circRNAs affect the chemoradiation resistance of cancer cells by regulating specific genes or pathways. For example, circ-0043632 regulates the proliferation, migration, and invasion of NSCLC, and AZD9291-resistant NSCLC may bypass the circ-0043632/miR-492/TIMP2 axis [143]. TIMP2 is critical for the regulation of ECM remodelling. In addition, the hypoxic microenvironment makes cancer cells more resistant to radiotherapy [148]. Hypoxia-induced cZNF292 can enhance the radioresistance of hypoxic hepatoma cells [103]. Notably, the presence of CSCs and angiogenesis are closely associated with therapeutic resistance [149–151]. CircRNAs can promote malignant progression and therapeutic resistance via modulating the CSC phenotype [116, 152]. Because of the close association of circRNAs with angiogenesis, targeting these related circRNAs may provide new insights into the reversal of cancer resistance through the regulation of angiogenesis.

With the increasing involvement of circRNAs elucidated by the study of chemoradiation resistance in cancer cells, as novel biomarkers, circRNAs have great potential for predicting the efficiency of chemoradiation and prognosis or for interfering with chemoradiation resistance as targets in clinical cancer therapy.

### CircRNAs as biomarkers in cancer

The application of biomarkers plays a vital role at all stages of cancer and has become one of the main approaches for cancer diagnosis, determination of prognosis and monitoring of progression. A competent biomarker should have good sensitivity, specificity, repeatability, stability and clinical utility [153]. The expression patterns and characteristics of circRNAs (universality, conservation, tissue/cell specificity, and stability) make them ideal candidates as biomarkers. In addition, circRNAs are enriched in human bodily fluids, such as saliva [154] and blood [155], making them easy to detect and making them suitable biomarkers for the detection of cancers, especially liquid biopsies.

As a diagnostic marker, plasma circ-0001785 had better diagnostic accuracy than CEA and CA15-3 in breast cancer patients, and its plasma level was closely related to the histological grade, the TNM stage and distant metastasis [156]. In addition, circ-0000181, which is downregulated in gastric cancer (GC), was shown to have high tissue specificity and plasma sensitivity, and the expression levels was significantly correlated with tumour diameter, distal metastasis and CA19-9, thus, circ-0000181 is a potential noninvasive diagnostic biomarker [157]. Analogously, circ-0000190 was also thought to be a potential diagnostic biomarker for GC, and its sensitivity and specificity are better than traditional markers CEA and CA19-9 [158]. Numerous studies have shown that circRNAs can be potential biomarkers for the early detection and screening of cancer.

As a metastatic marker, the expression of circ-0023988, circ-0008157 and circ-0030388 was elevated in high-metastatic melanoma compared with low-metastatic melanoma [159]. CircRNA-0001178 and circRNA-0000826 have been shown to be significantly differentially expressed between tissue samples from CRC patients with and without liver metastasis, so they may serve as a potential biomarker for liver metastases from CRC [160]. NSCLC tumour specimens exhibited higher circP4HB levels than paired healthy lung samples and were associated with metastatic disease [161]. HCC patients with high circ-ZNF652 expression were more prone to vascular invasion, intrahepatic metastasis, and distant metastasis [162].

As a prognostic marker, an elevated level of circHIPK3 was linked to poor prognosis in patients with glioma [163]. CircEPSTI1 and circKIF4A, which are significantly upregulated in triple-negative breast cancer (TNBC), were shown to be closely correlated with poor prognosis [164, 165]. CiRS-7 was found to be upregulated in CRC tissues was suggested to be an independent prognostic biomarker for the overall survival of patients with CRC [166]. Another study showed that osteosarcoma patients with high expression of circ-NT5C2 had a shorter overall survival (OS) and disease-free survival (DFS) than those with low expression of circ-NT5C2, which implies that circHIPK3 might be a new marker of prognosis in osteosarcoma [167].

In recent years, increasing evidence suggests that circRNAs can be not only clinical biomarkers for the early detection, diagnosis, metastasis and prognosis of cancer but potential therapeutic targets to increase anti-tumour response by regulating the TME. For example, we could target circRNAs to inhibit the expression of PD-L1, activate immune cells, prevent angiogenesis, decrease endothelial cell permeability, block the hypoxia pathway, destroy the ECM, and reverse cancer chemoradiation resistance. Research on these topics will provide new insights into targeted cancer therapy in the future.

## Conclusions

**Fig. 4:**
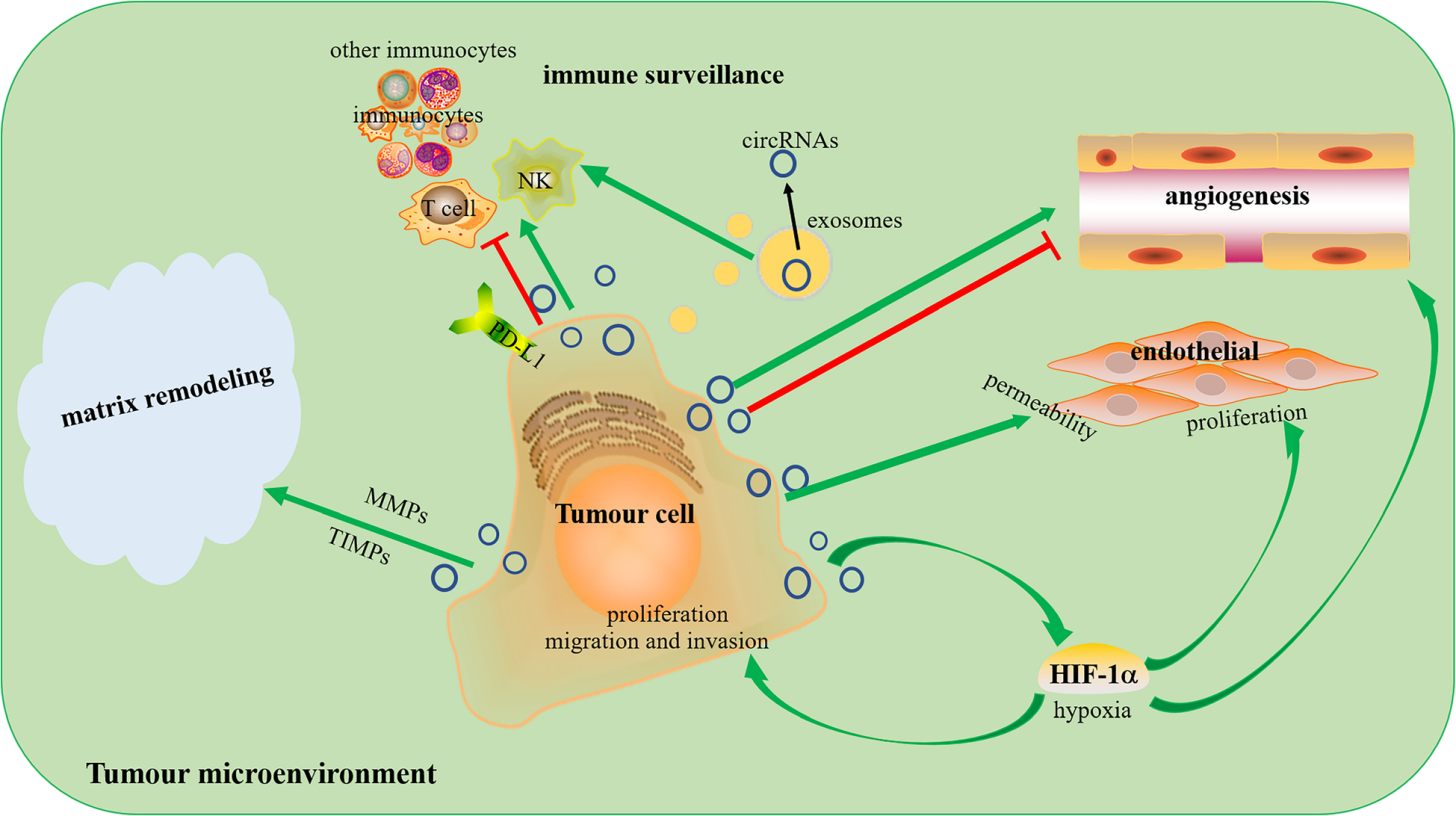
Roles of circRNAs in the TME: promote or inhibit the immune system and angiogenesis, improve the permeability of endothelial cells, and remodel the ECM. The green arrow indicates stimulatory modification, and the red “T” symbol indicates inhibitory modification

CircRNAs play multiple roles in the TME and can promote or inhibit the immune system and angiogenesis, improve the permeability of endothelial cells to promote cancer metastasis and cause ECM remodelling, which together supports tumour progression (Fig. 4).

Although there is growing evidence regarding the important roles of circRNAs in the TME, research on this topic is still in its infancy, and the physiological and pathological roles of circRNAs in the TME remain to be further explored. There are some problems that urgently need to be solved. First, unlike miRNAs, the study of circRNAs derived from stromal cells (such as CAFs, endothelial cells, pericytes and immune cells) in the TME is still in its infancy, especially regarding circRNAs derived from CAFs. Therefore, much research is needed to expand this field. Additionally, many studies have confirmed that exosomes play an essential role in pre-metastatic niches, and whether circRNAs are also involved in the formation of pre-metastatic niches needs to be determined. Last but not least, because of the heterogeneity of circRNAs in regulating the TME, the efficacy and safety of targeted therapies need to be tested.

In brief, we drew conclusions about the functions of circRNAs in the TME and further explored the enormous potential and unsolved problems of circular RNAs as potential biomarkers and therapeutic targets in clinical applications. We are convinced that circulating circRNAs might be used as liquid biopsies and noninvasive biomarkers for the early detection, diagnosis, and treatment of cancer in the future.

## Data Availability

Not applicable.

## Abbreviations

AR: androgen receptor
CircRNAs: Circular RNAs
CAFs: Cancer associated fibroblasts
CSC: Cancer stem cells; CRCCs: Colorectal cancer cells
CRC: Colorectal cancer
COL1A1: Collagen type I alpha 1 chain
DFS: Disease free survival
ECM: Extracellular matrix
EGFR: Epidermal growth factor receptor
EVs: Etracellular vesicles
EMT: Epithelial-mesenchymal transition
FDA: US Food and Drug Administration
GBM: Glioblastoma
GECs: Glioma-associated endothelial cells
HCC: Hepatocellular carcinoma
HUVECs: Human umbilical vein endothelial cells
HIFs: Hypoxia inducible factors
HREs: Hypoxia-responsive elements
HPSE: Heparanase
LncRNAs: Long ncRNAs
LSCC: Laryngeal squamous cell carcinoma
MiRNAs: MicroRNAs
MDSCs: Myeloid-derived suppressor cells
MREs: MicroRNA response elements
MMPs: Matrix metalloproteinases
ncRNAs: Noncoding RNAs
NK: Natural killer
NSCLC: Non-small-cell lung carcinoma
OS: Osteosarcoma
OS: Overall survival
PD-1: Programmed death 1
PD-L1: Programmed death-ligand 1
PC: Pancreatic cancer
PDAC: Pancreatic ductal adenocarcinoma
RBP: RNA-binding protein
SRSF1: Splicing factor Serine and Arginine rich splicing factor
SRY: Sex determining region Y
TME: Tumour microenvironment
Treg: Regulatory T cells
TAMs: Tumour associated macrophages
TILs: Tumour-infiltrating lymphocytes
TNBC: Triple-negative breast cancer
TICs: Tumour-initiating cells
TIMPs: Tissue inhibitors of metalloproteinases
TEXs: Tumour-related exosomes
USP7: Ubiquitin-specific protease 7
VEGFA: Vascular endothelial growth factor A
VM: Vasculogenic mimicry
